# Detection of *Babesia divergens *in southern Norway by using an immunofluorescence antibody test in cow sera

**DOI:** 10.1186/1751-0147-52-55

**Published:** 2010-10-06

**Authors:** Gunnar Hasle, Gunnar A Bjune, Dan Christensson, Knut H Røed, Anne C Whist, Hans P Leinaas

**Affiliations:** 1 Department of Biology, University of Oslo, P.O. Box 1050 Blindern, N-0316 Oslo, Norway; 2 Institute for Health and Society, Faculty of Medicine, University of Oslo, Norway; 3 Department of Virology, Immunobiology and Parasitology. National Veterinary Institute, Uppsala, Sweden; 4 Department of Basic Sciences and Aquatic Medicine, Norwegian School of Veterinary Science, Norway; 5 Department of Cattle Health Services, TINE Norwegian Dairy Association, Norway

## Abstract

**Background:**

The incidence of bovine babesiosis, caused by *Babesia divergens *(Apicomplexa: Piroplasmida) has decreased markedly since the 1930 s, but may re-emerge as a consequence of climate change and changes in legislation and pasturing practices. This is a potentially serious disease, with both economical and animal welfare consequences. Therefore, there is a need to survey the distribution of *B. divergens*.

**Methods:**

We tested sera from 306 healthy pastured cows from 24 farms along the southern Norwegian coast by using an indirect immunofluorescence IgG antibody test (IFAT). Fractions of seropositive cows were compared by calculating 95% CI.

**Results:**

The results of this test showed that 27% of the sera were positive for *B. divergens *antibodies. The fraction of antibody-positive sera that we detected showed a two-humped distribution, with a high fraction of positives being found in municipalities in the western and eastern parts of the study area, while the municipalities between these areas had few or no positive serum samples.

**Conclusions:**

Neither the farmers' observations nor the Norwegian Dairy Herd Recording System give an adequate picture of the distribution of bovine babesiosis. Serological testing of cows by using IFAT is a convenient way of screening for the presence of *B. divergens *in an area.

## Background

Though the incidence of bovine babesiosis is low in Norway, these pathogens have immense economic importance throughout the world, with the highest prevalence being found in the tropics [1]. The costs associated with this infection are associated with mortality, ill-thrift, abortions, loss of milk and meat production as well as with measures taken to control its spread [2]. *Babesia divergens *is the main cause of bovine babesiosis in northern Europe [3], although *B. major*, occurs in southeast England, Holland and the Friesian Islands in Germany [4]. *Babesia *species are intraerythrocytic protozoa that cause fever, haemoglobinuria (redwater) and anaemia in cattle that are exposed to the parasite as adults. Calves are relatively resistant to *B. divergens *[5,6] and exhibit mild or no effects of the disease, while infected adults may have a high mortality [7,8]. *Babesia *spp. can cause serious infections in humans who do not have a functioning spleen or who are immunocompromised as a result of immunosuppressive drugs, malignancy or HIV-infection [9]. The only case of human *B. divergens *diagnosed in Norway is a splenectomised veterinarian in Western Norway in 2007 (personal communication, Kristine Mørch, Haukeland University Hospital).

Cattle are the only natural vertebrate host for *B. divergens*. Reindeer and gerbils, and splenectomised individuals of other species may be infected experimentally. Sheep, wild cervids and rodents that occur in the area where it is distributed are all considered to be resistant to *B. divergens *[3]. However, this issue is controversial, as new studies indicate that roe deer and red deer may be infected by *B. divergens *[10,11]. The vector of *B. divergens *in Western Europe is *Ixodes ricinus *(Acari: Ixodidae) [3], which can parasitise a wide range of vertebrates [12]. Vertebrate hosts may act as vehicles for spreading *Babesia*-infected ticks, though only adult females of *I. ricinus *can become infected with *B. divergens *from cattle [13]. Transovarial and transstadial transmission of *B. divergens *occur in *I. ricinus *[14], and the infection can last for at least two generations [13]. Thus, these ticks may also represent a reservoir of the parasites, though only a small percentage of the larvae from the infected females usually carry the pathogen [13]. Each female of *I. ricinus *produces approximately 2,000 eggs [15], so there will be a correspondingly high mortality from one stage to the next in a stable tick population. Supposing a maximum 3 years generation time of *I. ricinus *and a maximum of three generations of parasite survival through transovarial transmission, the pathogen would, therefore, be expected to gradually disappear within a decade in areas where there are no vertebrate hosts present to transmit the infection to the ticks. After recovering from acute babesiosis, cattle may sustain a low level of parasitaemia for at least two years, which may be followed by the development of immunity to the parasite, without any detectable parasites in the blood [16]. Opsonising antibodies play an important role in protecting hosts against *B. divergens *infection, but the acquired immunity is not dependent on circulating antibodies, and *in vitro *tests have demonstrated a role of T-lymphocytes in protection against the disease. Antibody levels generally fall below the level of detection within six months after treatment [2]. The long-lasting host-parasite interaction results in the cattle acting as an effective reservoir of the parasites [17].

In Norway, the law does not mandate obligatory notification of bovine babesiosis, and no systematic study on the distribution of this parasite has been undertaken since the work of Thambs-Lyche from 1933-1940 where 1388 cases per year were reported [18]. One way of estimating the number of cases of this infection that exist today is by looking at sales of imidocarb, a veterinary medicine used to treat bovine babesiosis. Approximately 300 vials of 1200 mg imidocarb are sold per year in Norway (Bjørn Loe, Schering-Plough, personal communication), and this amount would be sufficient for treatment of a maximum of 600 individuals. Alternatively, data recorded at the Norwegian Dairy Herd Recording System (NDHRS) can be examined, since every cow in Norway is assigned an individual Cow Health Card on which all diseases are recorded by veterinarians or farmers and then reported to the NDHRS. This system has been in operation nationally since 1975 [19], and the health code and date of all disease treatment events are maintained in a central database. From 1996-2008, 121 cases of bovine babesiosis were reported in the NDHRS per year. Thus, both of these estimation methods indicate that the incidence of bovine babesiosis in Norway has fallen markedly since the 1930 s. This decrease coincides with, and may be explained by, a marked decrease in pasturing of cattle. In 1938, almost all of the 1.3 million cattle population in Norway were pastured regularly, whereas only 220,000 of the present 920,000 cattle population are pastured during the summer [20,21]. A decrease in bovine babesiosis has also been documented in Ireland. Gray et al. suggested that this might be due to a combination of several factors, such as an increase in average farm size and destruction of ticks' habitat by increased sheep pasturing. On the other hand, they suggested that the rate of clinical disease is low in western Ireland because of enzootic stability, i.e., the herds are naturally immune [22].

Bovine babesiosis is regarded as a limited problem in Norway, being confined to coastal areas north to southern Nordland county [23]. However, there may be a locally elevated risk of contracting babesiosis, which might be an argument against importing adult cows from inland localities where redwater does not occur and that, therefore, will not harbour any acquired immunity to the disease. In addition, changes in climate and pasturing practices could also lead to an increase in the incidence and distribution of bovine babesiosis. As the distributional range of ticks in Scandinavia expands [24], bovine babesiosis may be introduced into areas where livestock do not have a natural immunity to infection. We have no sound scientific data in support of an expansion of tick distribution in Norway, although this has been documented in Sweden [24]. Moreover, since 2004 all tie-stalled cattle in Norway have been required to be pastured for a minimum of 8 weeks during the summer [25], and this same legislation will also apply to cows in free-stalls by 2013, which could lead to an increase in bovine babesiosis. Because of these changes an updated map of the distribution of this parasite is needed for the purpose of better management. The distribution of *B. divergens *could be mapped by testing for the presence of the pathogen in ticks using PCR. Lundsett [26] tested 439 flagged ticks along the southern Norwegian coast and found only one tick that was positive for *B. divergens *using this method. Radzijevskaja [27] found no *B. divergens *in 91 ticks (16 adults, 75 nymphs) collected in Jomfruland, where we found that redwater is perceived to be a problem by farmers. Thus, testing ticks for *B. divergens *directly is both laborious and costly, and would require analysis of a very large number of ticks.

The aim of this study was to use a well-established indirect immunofluorescent antibody test (IFAT) to detect the presence of *B. divergens *antibodies in blood sera [28], and to evaluate this method as a means of mapping the distribution of the pathogen by comparing our results with information obtained either through reporting through the NDHRS or by interviewing the farmers.

## Materials and methods

**Figure 1:**
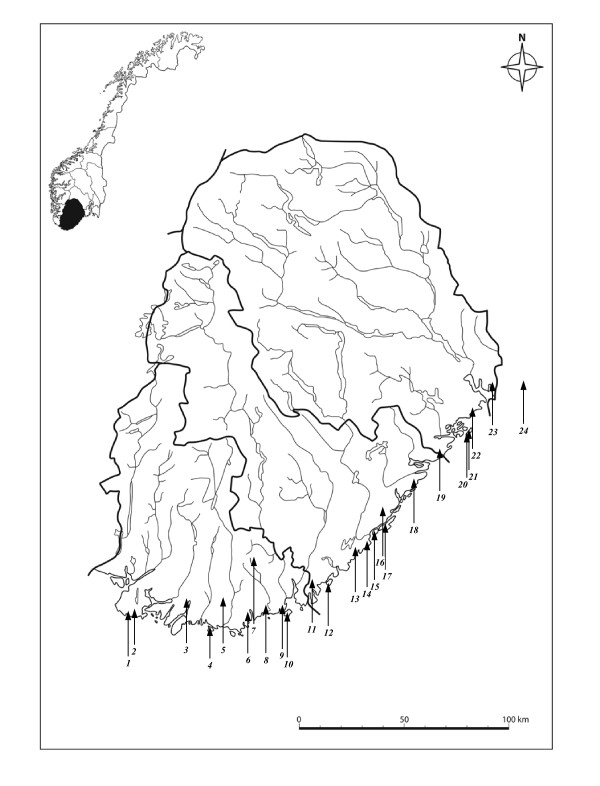
**Map of Vest-Agder, Aust-Agder and Telemark counties, with study localities numbered from west to east (Table 1)**. Farm number 24 is in Vestfold County. (Copyright, map basis: Cappelen Damm as.).

The study area consisted of farms with cows that were pastured in wooded areas within the previously established distribution of bovine babesiosis [29]. Twenty-four farms scattered along most of the southern Norwegian coast west of the Oslo Fjord (Figure 1) were included in the study. Farms using hillside or wooded areas for pasturing were identified with the help of local agricultural authorities. None of the farmers who were asked to participate in the study refused. All the farmers confirmed that ticks occur on their farms, and the cattle were pastured on the property. All of the 306 cows included in the study were healthy and at least one year old when tested. On one farm (Farm 23), all the cows had been purchased one year prior to the study from various inland localities and had been pastured for just one season at this farm. *I. ricinus *is distributed mainly near the coast in this part of Norway. The study included one inland farm approximately 30 kilometres from the sea (Farm 7) that was included because human Lyme borreliosis had been reported in this municipality, thus indicating the presence of ticks, according to the Norwegian Surveillance System for Communicable Diseases (MSIS) [30]. Blood samples were collected in May 2004 on farms 20 and 21, and samples were collected from all other sites in October and November 2005. The blood samples were stored at 4°C within a few hours after collection, and the serum portion of the samples was separated and frozen within 72 hours.

All of the sera were tested using an indirect immunofluorescent antibody test (IFAT) [28] for IgG as described by Christensson [31,32], and Christensson and Moren [33] with the following modifications: Antigen was prepared in 2002 from blood of a calf infected with *Babesia divergen*s with approx. 10% infected erythrocytes as described by Christensson [32]. The antiserum used was was FITC conjugated rabbit anti bovine IgG, produced by ICN Cappel, code 55280, lot 03683, diluted at 1/200 to give comparable readings with control sera used by Christensson and Morén [33]. Control sera were obtained from calves used for vaccine production in the year 2001 drawn before infection and four weeks after having showed acute parsitaemia. Negative control serum showed no or uncertain reaction at a dilution of 1/20 or higher. The positive control sera had an endpoint titre of 1/1280-1/2560. For each day of reading IFAT-slides a negative control at 1/40 and a positive control at 1/40, 1/160 and 1/1280 were included. As the purpose of the test was to identify seropositive/seronegative animals sera were read at dilutions at1/40 and 1/160. Slides were read blindly and scored by Christensson as having uncertain (+), positive (++) or strongly positive immunofluorescence (+++), at dilutions of 1:40 and 1:160. To minimise the risk of false positives, only sera with a minimum +++ score at a dilution of 1:40 were counted as positive.

Farmers were interviewed to determine if there had been cases of redwater on their farms and if they had experienced redwater in cows that were imported to the farm. Data on the cases of babesiosis in the included farms were obtained from the NDHRS.

To test the suitability of using PCR on full blood, we chose samples for a pilot study from four farms where redwater was common, according to the local farmers, and DNA from 100 μl from 20 samples of frozen EDTA-blood, and 25 samples of 100 μl blood clot, frozen after spinning and removal of the serum, were isolated in a spin-column, using DNeasy Blood & Tissue Kit (Qiagen), and eluated to 200 μl, according to the manufacturer's protocol. The isolation of DNA contained a lysis step and washing. Five μl of the eluate was run in *B. divergens *real-time PCR for 40 cycles with primers BdiF, BdiR and BdiT. The PCR was performed by Telelab (Skien, Norway), using an in-house method, as described by Lundsett [26]. The laboratory used a synthetic amplicon with the sequence of *B. divergens*, serially diluted in human DNA as a positive control. The reaction mix and human DNA was used as a negative control. The observed cutoff for detection was 30 *B. divergens *DNA copies, i.e. 15 to 30 individual cells, depending of whether they are asexual, diploid cells or sexual, haploid cells.

Exact confidence intervals for binomial proportions were calculated using the principles introduced by Clopper and Pearson [34] and implemented in R (R Development Core Team, 2008).

## Results

**Figure 2:**
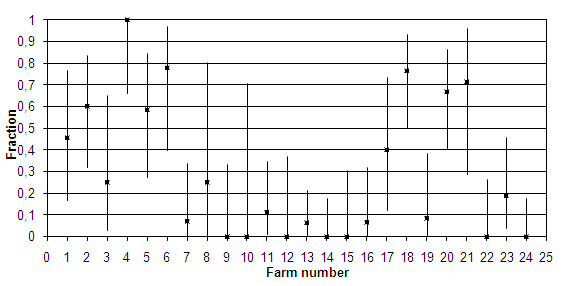
**Fraction of cows positive for *Babesia divergens *IFAT IgG antibodies at a titre of 1:40 (+++) or higher in 24 different farms along the southern Norwegian coast, arranged form west to east**. Error bars: 95% confidence intervals.

**Figure 3:**
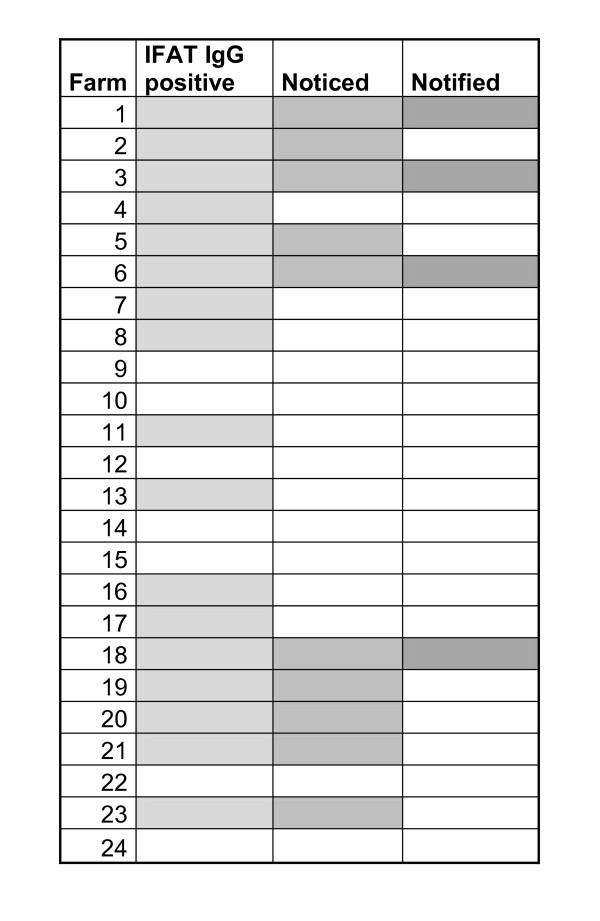
**Comparison of three sources of information for the occurrence of babesiosis on the farms in this study**. IFAT IgG positive: At least one cow positive for IFAT *Babesia divergens *IgG. Noticed: Farmers' statement that redwater occurs in cows on the farm or is detected when adult cattle are imported to the farm. Notified: Clinical cases registered on the diary cow health cards, compiled by the Norwegian Dairy Herd Recording System from 1996-2008.

Of the 306 sera that we tested, 84 (27%) had positive IFAT results. A high percentage of these positive results were found in the western and eastern ranges of the study area, and a much lower rate of positive test results was found in the middle range of the study area (Table 1; Figure 2). Farm 23 had 3 positive test results among the 16 cows that had been imported from inland localities one year before the study, indicating that there is a substantial risk of babesiosis in their present locality. The presence of *B. divergens *was confirmed by IFAT in a total of 17 of the 24 farms we tested. Farmers had observed redwater in only ten of the farms where *B. divergens *was detected, and only four of these cases of redwater had been recorded by the NDHRS (Figure 3). All of the cows on one of the farms in the study were *B. divergens*-antibody positive, though the owner had never seen any cases of redwater. We detected *B. divergens *antibodies in 17 of the 25 cows that we tested on Jomfruland, where Radzijevskaja [27] found no infected ticks.

**Table 1:** Municipality of the test localities in Figure 1 and test results of indirect immunofluorescence antibody tests (IFAT) for *Babesia divergens*.

Farm	Municipality	Neg	Pos^1^	N	% pos
1	Farsund	6	5	11	45
2	Farsund	6	9	15	60
3	Lyngdal	6	2	8	25
4	Mandal	0	9	9	100
5	Mandal	5	7	12	58
6	Søgne	2	7	9	78
7	Songdalen	13	1	14	7
8	Søgne	3	1	4	25
9	Kristiansand	9	0	9	0
10	Kristiansand	3	0	3	0
11	Lillesand	16	2	18	11
12	Lillesand	8	0	8	0
13	Grimstad	29	2	31	6
14	Grimstad	19	0	19	0
15	Arendal	10	0	10	0
16	Arendal	14	1	15	7
17	Arendal	6	4	10	40
18	Risør	4	13	17	76
19	Kragerø	11	1	12	8
20	Kragerø	6	12	18	67
21	Kragerø	2	5	7	71
22	Bamble	12	0	12	0
23	Porsgrunn	13	3	16	19
24	Larvik	19	0	19	0
Total		222	84	306	27

1. IFAT IgG titres scored as 1:40 (+++) or higher are defined as positive.

The PCR pilot study gave no positive results.

## Discussion

In Norway and Sweden the only cattle *Babesia *reported is *B. divergens *[35,36]. With regard to this and the strong reaction to the antigen used we assume that the seropositive animals were/had been infected with the species *Babesia divergens*. Our results demonstrate that testing of cattle for seropositivity to *B. divergens *is a far better method for mapping the distribution of this pathogen than using indirect methods, such as interviewing farmers or relying on the NDHRS. When it presents clinically, redwater is easily recognizable by farmers and veterinarians, and because prompt treatment is usually required to prevent deleterious effects of the disease, veterinarians often treat the disease without performing any laboratory tests. There are few data available on the attack rate of bovine *B. divergens *infections. Our data indicate that there are many subclinical cases of *B. divergens *infection, which is in agreement with previous studies on outbreaks [7,37] and in stable infected herds [38]. An extensive study of *B. divergens *seroprevalence was conducted in Northern Ireland, showing an overall seroprevalence of 31,8%[39], i.e., close to the overall seroprevalence in our limited material. A second study carried out in Northern Ireland [40] found consistent estimates when comparing results from a farm survey, a veterinary practise survey and seroprevalence data, with an estimated clinical incidence of 0,26% per year. The number of cases in the Agder counties, according to the NHDRS, is 18.4 cases per year in a population of ca. 10400 dairy cows (Statistics Norway, http://www.ssb.no/emner/10/04/10/jt1999/tab-2001-04-03-07.html, Jordbrukstelling 1999), which would give an incidence of 0.18% per year. Our results indicate an incomplete registration of cases of redwater in the NHDRS, possibly because veterinarians are not always consulted e. g. during the dry period, in mild cases of redwater, or that the farmers fail to observe redwater while the cows are out at pasture. The farms that we included in our study were not randomly selected, but were chosen because the pastures were in wooded areas, and were situated near the coast in the distribution area of *I. ricinus *in Norway. They would therefore be expected to have more babesiosis than average farms in the same counties.

Because cows are parasitised by hundreds of ticks in the course of a season, and a single bite from an infected tick is sufficient for transmission of *Babesia*, [41] cows are likely to contract *B. divergens *if it is present in their pasturing areas. The screening of cows for *B. divergens *infection would therefore be expected to be a sensitive method for detecting the presence of the parasite in a locality, if testing is performed at a time of the year when *Babesia-*antibodies are at the highest. Serum samples that we collected on Jomfruland in May were not directly comparable to those that we collected in October and November, as the May samples could either contain persistent antibodies from the previous year, or there might be early infections from the same year. The mean temperature April 1^st^-15^th ^was 5.3°C, and no temperatures of below 0°C were recorded (The Norwegian Meteorological Institute), which means that tick questing may well have occurred during this period. With an incubation time of 1-3 weeks [3], seroconversions during May 2004 would be expected to occur. As we tested only once for each locality we did not demonstrate the seasonal and yearly variation of antibodies described by l'Hostis et al. [38]. Further studies are needed to decide which month would be optimal for detecting the presence of *B. divergens *in a locality along the Southern Norwegian coast. However, ticks are still parasitizing the cows in October and November and these months would therefore be expected to be a good choice for detecting *B. divergens *antibodies.

The sensitivity of serologic testing for detecting *B. divergens *will depend on the cut-off level that is set for a positive score on the test. At a cut-off level of 1:40 (++) the sensitivity and specificity of an individual antibody test are reported to be 100% and 97%, respectively [32]. Setting the cut-off value at this level would, therefore, likely result in the detection of a few false positives due to non-specific cross reactivity. This problem is illustrated by our results on Farm 24, where only one cow was found to be positive at the detection level of 1:40 (++), and there were no positive tests at more stringent detection levels. This result could represent either a false positive or a low titre in a cow that was infected a long time ago. Because the aim of this study was to be able to detect the present occurrence of *B. divergens *at a particular locality, a high sensitivity for detecting the pathogen on a given farm is desirable, and the number of cows tested is crucial. By testing a median of twelve cows per locality, we were able to achieve a much higher sensitivity for detecting *B. divergens *on a given farm than farmers' observations and the existing NDHRS can provide. At all the farms where samples with 1/40(+++) were detected there were also samples positive at 1/160, indicating that these are real positives. Therefore, by setting a cut-off level of 1:40 (+++) for defining a case of seropositivity for *B. divergens*, antibody testing should result in a specificity of nearly 100%, unless cross-reacting *Babesia *spp. are occurring and, consequently, the risk of falsely concluding that *B. divergens *occurs on a farm will be small. The related species *B. capreoli *cause babesiosis in roe deer and red deer [42], and roe deer may also be infected by the newly discovered *Babesia *sp. EU1 [43]. These parasites cannot be serologically distinguished from *B. divergens*. They cannot give clinical infection in cattle, but there is a possibility that a subclinical infection may cause seroconversion [44], although Schmid et al. [45] found no seropositive cows in an area in which ticks positive for these two non-bovine *Babesia *species were found. It is therefore unlikely that these *Babesia *species would influence the number of seropositive cows in this study significantly. There are no published studies on these *Babesia *species in Norway, but a Swedish study suggested that babesiosis caused by *B. capreoli *is very rare in Sweden [46].

An alternative to antibody testing is to test directly for the presence of the pathogen in cattle blood samples. Calder et al. [47] found an approximately 80% sensitivity for detecting *Babesia bovis *by PCR in steers, up to 300 days after experimental infection. The method these investigators used required a concentration step involving ultracentrifugation of haemolysed blood. We considered this to be too laborious a method to be useful as a field assay. We did attempt direct PCR to detect *B. divergens *without performing the concentration step in 30 samples from areas where we found the highest incidence of *B. divergens *by IFAT, but none of these samples were found to be *B. divergens*-positive by this method. Cultivation of *Babesia *in cell culture, which enables detection of *Babesia *at a level of 10 parasites per 1 ml of blood [48], is another possibility for mapping the distribution of this parasite, but it is not feasible to use this method when sampling is being carried out in scattered locations. For our purposes, therefore, we found that antibody screening was a much more convenient method for assessment of the occurrence of *B. divergens *in a locality than any of the other methods that are available for detecting this pathogen. Gerbil-derived antigen is found to be equally specific to *B. divergens *obtained from cattle [49], and could be a cheaper alternative in future studies.

In the communities on the coast of southern Norway where cows are pastured, the animals are confined to the farms on which they are kept. Consequently, testing cows for the presence of *B. divergens *infection should provide results that are specific to a given locality, as opposed to performing serological testing on other hosts of tick-borne pathogens, such as wildlife, dogs or humans. Because *B. divergence *is unlikely to survive for more than a decade in regions where cattle are not pasturing and cattle is the only host for *B. divergens *at the Southern coast of Norway, testing cow sera appears to be an effective method for mapping *B. divergens *over the area of distribution of *I. ricinus*. The same is not the case if using cattle as sentinel animals for serological testing for other tick-borne pathogens, such as *Anaplasma*, *Borrelia *or the TBE virus, that infect a wider range of hosts.

Malandrin et al. [48] found a drop in IFAT antibody titre from 320, 320 and 1280 to 80, 80 and 320 respectively in samples from three cows taken 6 and 9 months after acute babesiosis, indicating an antibody duration of more than a year, but much shorter than the cows' lifespan. Sahibi et al. [50] found no significant cumulative effect of cow age on the presence of *Babesia-*antibodies. This is consistent with a short duration of antibodies in the bloodstream after infection, meaning that detection of antibodies indicates a recent infection, as is illustrated by the seasonal variation of *B. divergens*-antibodies that was found by l'Hostis et al. [38], indicating repeated infections during the season. This implies that the lifetime risk of acquiring bovine babesiosis is higher than the current rate of infection that was determined in the study we present here.

Our IFAT data indicate that there are two areas along the southern Norwegian coast in which bovine babesiosis is highly endemic, consisting of one western area (Lista-Mandal) and one eastern area (Kroger-Risør) (Figure 1, Table 1). This uneven distribution was not reported by Thambs-Lyche in a study carried out along the same part of coastal Norway [29]. For other *Babesia *species, it has been shown that reduction of the incidence of tick bites can bring the reproduction rate of the parasite below 1, indicating that it could be possible to eradicate the parasite [41,51]. Our results indicate that, in the area from Sandaled to Arundel, which is within the distribution area of *I. ricinus *and is an area where cattle are pastured in a natural setting, *B. divergens *occurs at very low frequencies or not at all. In fact, the disease associated with this pathogen has virtually disappeared since the 1930 s, when Thambs-Lyche reported babesiosis in this area. This seems promising with regard to the possibility of eradicating this disease. An attempt to eradicate the disease would require the implementation of control measures over its entire distribution because wild hosts can spread infected ticks. Cervid animals are the most important hosts for adult ticks [52]. Red deer, roe deer and moose have yearly migratory ranges of 200, 100 and 50-60 kilometres respectively [53], and Cervid animals, therefore, have the potential for transporting large numbers of ticks over long distances. Furthermore, birds can transport ticks across geographical barriers. In a recent study, 7.3% of northward migratory passerine birds were found to carry one or more ticks [54], so, in a situation where cows are pastured in an area that is free of *B. divergens*, or where there is an unstable population of the pathogen, *B. divergens *could conceivably be introduced by birds.

## Conclusions

At present, bovine babesiosis is a limited animal health problem in Norway. The most obvious possible cause of the decline in incidence since the 1930 s is changes in the use of pastures. Changes in legislation leading to increased use of wood pasturing may reverse the decline in incidence, and we may also see a climate-related increase. An increased incidence of *B. divergens *in cattle could have important economic and animal welfare consequences, and further studies are needed to evaluate whether it would be cost effective to implement preventive measures against the spread of this pathogen. Antibody testing of pastured cows is a simple way of mapping the distribution of the pathogen.

## Competing interests

The authors declare that they have no competing interests.

## Authors' contributions

GH prepared the fieldwork, interviewed the farmers, performed all the blood sampling and wrote the main part of the paper. GB, KHR and HPL provided valuable and significant contributions to the writing of the paper. DC headed the laboratory work, and performed all the microscopy of the slides in the immunofluorescence antibody test. Furthermore, he contributed significantly to the writing of the paper. ACW contributed with data from the Norwegian Dairy Herd Recording System, and also contributed significantly to the writing of the paper. All authors read and approved the final manuscript
